# Co-regulation of *Sox9* and *TGF*β*1* transcription factors in mesenchymal stem cells regenerated the intervertebral disc degeneration

**DOI:** 10.3389/fmed.2023.1127303

**Published:** 2023-03-17

**Authors:** Shumaila Khalid, Sobia Ekram, Faiza Ramzan, Asmat Salim, Irfan Khan

**Affiliations:** Dr. Panjwani Center for Molecular Medicine and Drug Research, International Center for Chemical and Biological Sciences, University of Karachi, Karachi, Sindh, Pakistan

**Keywords:** intervertebral disc degeneration, mesenchymal stem cells, cartilage, transcriptional regulators, regeneration, chondrocyte

## Abstract

**Background:**

Intervertebral disc (IVD) shows aging and degenerative changes earlier than any other body connective tissue. Its repair and regeneration provide a considerable challenge in regenerative medicine due to its high degree of infrastructure and mechanical complexity. Mesenchymal stem cells, due to their tissue resurfacing potential, represent many explanatory pathways to regenerate a tissue breakdown.

**Methods:**

This study was undertaken to evaluate the co-regulation of *Sox9* and *TGF*β*1* in differentiating human umbilical cord mesenchymal stem cells (hUC-MSC) into chondrocytes. The combinatorial impact of *Sox9* and *TGF*β*1* on hUC-MSCs was examined *in vitro* by gene expression and immunocytochemical staining. In *in vivo*, an animal model of IVD degeneration was established under a fluoroscopic guided system through needle puncture of the caudal disc. Normal and transfected MSCs were transplanted. Oxidative stress, pain, and inflammatory markers were evaluated by qPCR. Disc height index (DHI), water content, and gag content were analyzed. Histological examinations were performed to evaluate the degree of regeneration.

**Results:**

hUC-MSC transfected with *Sox9*+*TGF*β*1* showed a noticeable morphological appearance of a chondrocyte, and highly expressed chondrogenic markers (*aggrecan, Sox9, TGF*β*1, TGF*β*2*, and type II collagens) after transfection. Histological observation demonstrated that cartilage regeneration, extracellular matrix synthesis, and collagen remodeling were significant upon staining with H&E, Alcian blue, and Masson's trichrome stain on day 14. Additionally, oxidative stress, pain, and inflammatory markers were positively downregulated in the animals transplanted with *Sox9* and *TGF*β*1* transfected MSCs.

**Conclusion:**

These findings indicate that the combinatorial effect of *Sox9* and *TGF*β*1* substantially accelerates the chondrogenesis in hUC-MSCs. Cartilage regeneration and matrix synthesis were significantly enhanced. Therefore, a synergistic effect of *Sox9* and *TGF*β*1* could be an immense therapeutic combination in the tissue engineering of cartilaginous joint bio-prostheses and a novel candidate for cartilage stabilization.

## Introduction

The prevalence of disability and impairment of connective tissues such as the meniscus, knee joint cartilage, and nucleus pulposus of the intervertebral disc (IVD) is due to increased age, physical injury, athletic activities, and progressive changes. Several diseases are responsible for cartilage degradation including rheumatoid arthritis (RA), and osteoarthritis (OS) which account for about 20%–50% of musculoskeletal disabilities in adults as reported in the Center for Disease Control and Prevention (CDC) statistics (1). Another substantial cause of disability is lower back pain (LBP) which accounts for 80% of disabilities worldwide. The cause is idiopathic, however, due to some reasons, it is disc degeneration (2). The cartilage of the intervertebral disc shows aging and degenerative changes earlier than any other body connective tissue. The IVD is a multifaceted joint that allows flexion within the spine while functioning as a shock absorber. The major purpose is to permit 3D motion, preclude disproportionate motions, and uphold sustained mechanical stability (3–5). IVD comprises three main components: the central nucleus pulposus (NP), lamellar concentric rings of the annulus fibrosis (AF), and the cartilaginous end plate (CEP) (6, 7). Being the largest avascular tissue in the body, its regeneration, and repair are poor resulting in fibrosis with biomechanical potential that is greatly superior to normal tissue. Additionally, it is an annurel which comprises nerve extensions that are limited to the periphery of the disc (8, 9).

Due to the avascular environment, nutrient transportation and metabolic products are exclusively based on diffusion across the cartilaginous end plate and with the matrix (10, 11). If nutrient supply to cells is halted and removal of waste products is interrupted, then the accumulation of waste products lingers in the extracellular matrix inhibiting the health of chondrocytes (12, 13). In IVDs, the oxygen gradient reduces toward the central nucleus pulposus (14). Therefore, IVD chondro-progenitors or notochordal cells in different regions are subjected to different oxygen concentrations, effect of their migration potential by hypoxia varying at different degrees (15). The substantial percentage of glycosaminoglycans (GAGs) in the tissue is significantly important to maintain disc height, compressive properties, swelling pressure, and the synthesis of collagen type II. Approximately, a 65% reduction of proteoglycans is responsible for disc degeneration compared to healthy tissue (16). The cellular environment is greatly affected during disc degeneration. A striking difference is observed in the chondrocyte phenotype to the fibrotic phenotype in the central nucleus pulposus (17). Additionally, IVD size and shape are distorted by a change in water content which hinders the ability to bear loads (18).

Therapeutic strategies such as discectomy, microfracture, and autologous chondrocyte transplantation (ACT) alleviate the discomfort caused by disc degeneration and restore mobility, but these therapies do not regenerate the tissue architecture that mimics its native form (1). Therefore, mesenchymal stem cell (MSC) in regenerative medicine has emerged to reduce the chances of the degree of disease invasiveness and donor-side morbidity potentially resulting in superior regenerative potential (19, 20). It is the best option with the huge chondrogenic potential and less hypertrophic differentiation. However, the proper management of its differentiation potential is one of the keys to regenerative medicine (21). With advances in biological treatments, MSCs pretreated with pharmaceutical leads, small molecules, growth factors, and peptides have been extensively studied for the regeneration of degenerated IVDs. But these approaches were intended to be effective for the early stage of degeneration. However, the caveat requires repeated delivery of doses for maximum benefit, as the proper effect wears off with cell depletion over time (22, 23). Inducing differentiation in MSCs *via* transfecting chondro-specific genes before transplanting them into IVDs is a unique concept that significantly favors improving the microenvironment for regeneration. One of the greatest benefits of utilizing stem cell-based gene therapy is that it can possess a long-term effect. Nonetheless, these cells need to be examined for effectiveness and safety (24, 25).

Gene transfection of *TGF-*β*1* in MSCs has illustrated a twofold upregulation in proteoglycans (PGs) synthesis and transplanted IVDs showed a significant increase in *TGF-*β and PGs synthesis, which was consistent over the period of the observation. This outcome was considerably for longer duration than that with the preconditioning strategy (26). Besides, *TGF-*β, *Sox9*, and *LMP-1* transfection were also effective in reducing IVD degeneration. Similarly, murine MSCs were genetically modified using the *TGF*β*2* gene which significantly downregulated the hypertrophic profile and increased proteoglycans, whereas *BMP2* led to maintained chondrogenesis when transplanted in IVD (27). Furthermore, collagen production was increased in mouse models of osteoarthritis imperfecta when BM-derived MSCs were genetically modified using procollagen alpha 2 (28). In a study, sox-trio genes including *Sox9, Sox6*, and *Sox5* were transfected into the MSCs, followed by transplantation which significantly enhanced the chondrogenesis and halted the hypertrophic mechanisms when compared with the non-transfected MSCs (29).

The current study is designed to check the effect of overexpression of chondrogenic regulators in the differentiation of MSCs to notochordal lineage and evaluate their role in the regeneration of IVD degeneration. Nevertheless, none of the studies has reported the co-delivery of *Sox9* and *TGF*β*1* in human umbilical cord-derived-MSCs for the regeneration of the degenerated disc. Hence, it was hypothesized that human umbilical cord-derived mesenchymal stem cells (hMSCs) can be transfected effectively *via* electroporation by co-expressing *Sox9* and *TGF*β*1*. The transfected cells should show increased production of collagen type 2 *in vitro* compared to the non-transfected cells. Moreover, transplantation of transfected hMSCs directly into the rat model of the degenerated disc should reverse the pathological changes with increased GAGs, and water content *in vivo*.

## Methodology

### Culture of human umbilical cord tissue

All protocols performed were in accordance with the ethical committee protocol no. IEC-009-UCB-2015 of Dr. Panjwani Center for Molecular Medicine and Drug Research, University of Karachi. Umbilical cord tissue was collected with the donor's informed consent during full-term childbirth. Before parturition, puerpera tested negative for HBV, HCV, and HCMV. Umbilical cord tissue samples were collected in sterile anticoagulant and transferred to the laboratory within 2 h. For the human umbilical cord tissue (hUC)-MSC primary culture, the explant method opted as reported in a prior study (30). Briefly, arteries and veins from tissue were removed to expose Wharton's jelly. Next, umbilical cord tissue was chopped into small fragments and transferred into T-75 cm^2^ (Nest, China) containing Dulbecco's Modified Eagle's Medium (DMEM) supplemented with 1 mM sodium pyruvate, 1 mM L-glutamine, 10% fetal bovine serum, and 1% penicillin-streptomycin. The culture flask was placed in an incubator set at 37°C, 5% (v/v) CO_2_, and 95% humidity. The medium was replaced after every 3 days until the cells were released from the explant and reached 50% confluence. The explanted were removed and re-cultured in another cultured flask. Cells were passaged when reached 70%−80% confluence. The 3rd till 5th passage cells were utilized in all the experiments.

### Identification of mesenchymal stem cells

#### Morphological assessment

The morphological appearance was observed during passages under a phase contrast microscope (Nikon Ti-2, Japan).

#### Osteogenic, chondrogenic, and adipogenic differentiation

To examine the differentiation ability of hUC-MSCs, osteogenic, chondrogenic, and adipogenic media was prepared. MSCs at passage 3 were cultured in six well-plate and incubated for 24 h in an incubator set at 37°C, 5% (v/v) CO_2_, and 95% humidity. After monolayer formation, MSCs were incubated in an osteogenic induction medium (1 μM Dexamethasone, 10 μM Insulin, and 200 μM Indomethacin), chondrogenic induction medium (1 μM dexamethasone, 10 ng insulin, and 20 ng TGFβ1 and 100 μM ascorbic acid), and adipogenic induction medium (1 μM Dexamethasone, 10 μM Insulin, and 200 μM Indomethacin) till day 21. Finally, differentiated cells were evaluated through Alizarin Red S, Alcian blue, and Oil Red O staining, respectively.

#### Flow cytometric analysis

MSCs surface markers were evaluated by flow cytometry. Briefly, cells at passage three were washed with FACS buffer and stained with anti-CD105, anti-Vimentin, anti-CD45, and Anti-CD90 for 1 h at RT in the dark. Then, they were incubated with the secondary antibody Alexa fluor 546 followed by washing with buffer and finally resuspended in FACS buffer for analysis on flow cytometer (BD FACS Celesta, Becton Dickinson, USA).

#### Immunocytochemical staining

The isolated population of cells from the primary culture of human umbilical cord tissue was grown on coverslips, fixed with 4% Paraformaldehyde (PFA) at room temperature (RT) for 10 min, and permeabilized with 1% Triton X-100. Blocking was achieved using 2% bovine serum albumin (BSA) and 0.1% Tween-20 in PBS for 15 min at RT, and incubating with the anti-primary antibodies against CD29, Vimentin, CD73, CD105, CD117, Lin28, Stro-1, CD45, and HLA-DR at the recommended dilution at 4°C overnight. Washed with PBS and incubated with secondary antibodies Alexa fluor 546 at dilution of 1:200 for 1 h at 37°C. Counterstain with DAPI, and Philloidin Alexa fluor 488 for nuclei and F-actin visualization. Lastly, cells were mounted with an aqueous mounting medium and were visualized under the fluorescent microscope (Nikon NiE, Japan).

### Reporters construct and isolation

Vector constructs for pcDNA3.1 HA-rn*Sox9* (62972, Addgene, USA), and TGFB1_pLX307 (98377, Addgene, USA) as *Escherichia coli* stab cultures were purchased from Addgene (www.addgene.org). Bacteria were grown in lauria broth and plasmid isolation was performed following the standard plasmid DNA isolation kit. Isolated plasmids were quantified by Nano-Drop spectrophotometer (NanoDrop ND-1000 Spectrophotometer; Thermo Fisher Scientific, Inc., Wilmington).

### Transient transfection

All the experiments were undertaken in a triplicate manner. MSCs were cultured at a density of 1.5 × 10^6^ in T-75 cm^2^. The subsequent morning, hMSCs were trypsinized and resuspended in sterile R buffer containing 30 μg total plasmid DNA of *Sox9*, and *TGFB1* and electroporated at an optimized program comprising input voltage of 1,200 volts; input pulse width of 10 ms and input pulse number of one pulse with Neon Transfection System (ThermoScientific, USA). The transfection medium and the plasmids, along with cells, were incubated at 37°C for 48 h, and then the medium was replaced with complete DMEM. Transfected hMSCs were observed and collected after 48 h of transfection to evaluate the transfection efficiency. Non-transfected MSCs groups cultured in DMEM, and chondro-induction medium (CM) were included as a negative, and positive control, respectively.

### Characterization of genetically modified MSCs

Transfected MSCs after 48 h, 7, 14, and 21 days were observed based on their morphological appearance and at the translational and transcriptional levels.

#### Morphological assessment

Transfected hMSCs were fixed with 4% PFA and permeabilized by 1% Triton X-100 for 10 min at RT. Blocking was achieved by incubating cells with 2% bovine serum albumin (BSA) and 0.1% Tween-20 in PBS for 30 min at RT. Finally, cells were stained with Alexa fluor 488 phalloidin molecules, and DAPI to visualize F-actin and nuclei. Mounting was performed using an aqueous mounting medium, and transfected hMSCs were visualized under the fluorescent microscope (Nikon NiE, Japan).

#### Translational analysis

After 48 h, 7, 14, and 21 days, transfected and non-transfected hMSCs were fixed, permeabilized, and blocked by a 2% BSA solution. Then, the cells were treated with anti-AGGRECAN, anti-SIX1, anti-SOX9, anti-STRO-1, anti-TGFβ1, anti-TGFβ2, and anti-COLII. Alexa fluor 546 secondary antibodies were used to detect primary antibodies, followed by 2 h of incubation with phalloidin labeled Alexa flour 488. Nuclei were visualized with DAPI. Mounting was performed to observe under the fluorescent microscope (Nikon NiE, Japan).

#### mRNA extraction and qRT-PCR

Cells were detached and pelleted by centrifugation (1,000 rcf and 8 min), and the Trizol method was opted to isolate and purify mRNA from the pallet followed by the previously illustrated method (30). Briefly, Trizol was added to the pallet for 10 min at RT followed by adding 200 μl chloroform for 10 min at RT and centrifuged at 12,000 rpm for 15 min. The aqueous layer was separated out and 1 mL of absolute ethanol was added, the suspension was kept at −20°C overnight. On the next day, the suspension was centrifuged at 12,000 rpm for 30 min for RNA pallet formation, followed by 70% and 100% ethanol wash and resuspension in 20 μl nuclease-free water and stored at −20°C. Extracted RNA was quantified by taking the absorbance at 260 and 280 nm using a spectrophotometer (NanoDrop ND-1000 Spectrophotometer; Thermo Fisher Scientific, Inc., Wilmington). Complimentary DNA was synthesized *via* cDNA reverse transcription kits (K1622, ThermoScientific, USA). One microgram of RNA was added to a 5 μl RT master mix containing dNTP mix, 5 × Reaction buffer, random hexamer, and 1 μl of reverse transcriptase. The reaction was run at 42°C for 60 minutes and 72°C for 5 min. The expression of *aggrecan, type II collagen* (*ColII*), and *SOX9, TGF*β*1, BMP2*, and *Six1* were calculated (Table 1). Beta Actin (β-Actin) was used as an endogenous control. The ΔCt (change in cyclic threshold) which is the change in cyclic threshold was determined by subtracting the Ct for β-Actin from the Ct value for each targeted gene. The ΔCt was normalized to the endogenous control to determine ΔΔCt. Relative fold change was obtained using the 2^−ΔΔCt^ method.

**Table 1:** Primer sequences used in study with their annealing temperature.

**Genes**	**Primer sequences (5^′^-3^′^)**	**Annealing temperature (°C)**
*GAPDH*	(F) 5′-CACCATGGGGAAGGTGAAGG-3′	58
	(R) 5′-AGCATCGCCCCACTTGATTT-3′	
*β-actin*	(F) 5′-CACTGGCATCGTGATGGACT-3′	58
	(R) 5′-TGGCCATCTCTTGCTCGAAG-3′	
*Sox9*	(F) 5′-CATCTCCCCCAACGCCA-3′	58
	(R) 5′-TGGGATTGCCCCGAGTG-3′	
*Six1*	*(*F) 5′-CTCCAGTCTGGTGGACTTGG-3′	58
	(R) 5′-AGCTTGAGATCGCTGTTGGT-3′	
*BMP2*	(F) 5′-AGCTGGGCCGCAGGA-3′	58
	(R) 5′-TCGGCTGGCTGCCCT-3′	
*ACAN*	(F) 5′-AATCTCACAATGCCACGCTG-3′	58
	(R) 5′-GAGGCTGCATACCTCGGAAG-3′	
*TGFβ1*	(F) 5′-CAAGGCACAGGGGACCAG-3′	58
	(R) 5′-CAGGTTCCTGGTGGGCAG-3′	
*ColII*	(F) 5′-CCCGGCACTCCTGGC-3′	58
	(R) 5′-GGAGGGCCCTGTGCG-3′	
*COX2*	(F) 5′-TGACTTTGGCAGGCTGGATT-3′	58
	(R) 5′-ACTGCACTTCTGGTACCGTG-3′	
*MMP-13*	(F) 5′-ACAGCAAGAATAAAGACTGTGCG-3′	58
	(R) 5′-CACATCAGTAAGCACCAAGTGTC-3′	
*ADRB2*	(F) 5′-TTATCGTCCTGGCCATCGTG-3′	58
	(R) 5′-GAAGTCCAGAACTCGCACCA-3′	
*Substance P*	(F) 5′-ACCTCCCATGATGACCCTGA-3′	58
	(R) 5′-TGCTGCAGTTTCGGTACACT-3′	
*COMP2*	(F) 5′-TGGTTCGAAACCCAGACCAG-3′	58
	(R) 5′-AACACCATCACCATCGCTGT-3′	
*CXCL2*	(F) 5′-GCGCCCAGACAGAAGTCATA-3′	58
	(R) 5′-CAGGTACGATCCAGGCTTCC-3′	
*YKL-40*	(F) 5′-GGTGCTACGAGAAGCTGTCA-3′	58
	(R) 5′-TTCCACTCCGATGTGCTGAG-3′	
*SOD1*	(F) 5′-ATTCACTTCGAGCAGAAGGCA-3′	58
	(R) 5′-CCTTTCCAGCAGCCACATTG-3′	
*PRDX1*	(F) 5′-TATCAGATCCCAAGCGCACC-3′	58
	(R) 5′-GTCCAGTGCTCACTTCTGCT-3′	
*GPX1*	(F) 5′-CCTCAAGTATGTCCGACCCG-3′	58
	(R) 5′-GATGTCGATGGTGCGAAAGC-3′	

#### Stemness marker analysis of transfected and non-transfected hMSCs

Transfected hMSCs after 48 h, 7, 14, and 21 days of culture in DMEM, and chondro induction medium (CM) were analyzed for the expression of stromal-specific maker Lin28. The transfected hMSCs showed significantly negative expression of Lin28 in contrast to the control group. The cellular cytoskeleton was stained with phalloidin molecules of Alexa Flour 488, and nuclei were visualized with DAPI. The images were taken using a fluorescent microscope (Nikon NiE, Japan) at 20 x magnification.

### Experimental animals

Sixty Wistar rats ranging in age from 3 to 6 months weighing ~250–350 g were obtained from the animal laboratory of Dr. Panjwani Center for Molecular Medicine and Drug Research (PCMD), University of Karachi, Pakistan. Animal studies were carried out under local ethical approval protocol number (#20170051) from the Institutional Animal Care and Use Committee of the International Center of Chemical and Biological Sciences (ICCBS).

### Animal groups

In this study, the animals were randomly categorized into four groups: one non-punctured normal group (*n* = 15), one punctured degenerated group (*n* = 15), one punctured transplanted with hMSCs group (*n* = 15), and one punctured transplanted with transfected hMSCs group (*n* = 15), respectively. The animals placed in the non-punctured group served as control subjects. Animals were maintained at a temperature of 26–30°C, relative humidity of 75%−85%, and 12-h light/dark cycle. Before the experiments, all the animals were given a few days to acclimatize to the new cages and environment.

### Needle puncture tail disc model development

The surgical procedure was performed as previously reported (30, 31). Briefly, the animals were anesthetized by a combined dose of ketamine hydrochloride (60 mg/kg) and xylazine hydrochloride (7 mg/kg) injection in the peritoneum. The rat tail was disinfected with 70% ethanol, and animals were placed in a prone position. Under the fluoroscopic guided system, percutaneous needle puncture was induced with an 18 G needle at the caudal disc: Co5–Co6, Co7–Co8, and Co9–Co10. The Co7–Co8, and Co9–Co10 served as positive and negative controls. The needle was inserted with full penetration until it reached the nucleus pulposus or the middle of the disc, parallel to the cartilaginous end plate, and at 90° of the skin, rotated 180° and held for 10s. After inducing degeneration, the needle was taken out, and the animals went through the standard postoperative protocols.

### Cellular labeling and transplantation

Normal and transfected hMSCs were trypsinized and incubated with DiI cell labeling dye (V-22885, Vybrant^®^ DiI cell-labeling solution, Invitrogen, USA) for 20 min according to the standard protocol. The cells were washed with PBS and finally resuspended in 50 μL of sterile PBS. The labeled cells were then transplanted in rat Co5–Co6 immediately after inducing degeneration. Two weeks of post-transplantation, the rats were euthanized and Co5–Co6, Co7–Co8, and Co9–Co10 discs were precisely harvested and demineralized in 11% formic acid for 1 h for histological analysis. IVD tissue was placed in molds containing frozen sectioning media called optimal cutting temperature (OCT) media (Surgipath, FSC22, Leica Microsystems, USA) and allowed at RT for 2 h to remove any bubbles. Finally, the molds were stored at −20°C.

### Histological and immunohistochemical assessment

Cryosectioning of frozen blocks was accomplished using a cryostat machine (Shandon, Thermo Electron Corporation, UK). A sharp cutting blade was used to take 8 μm thick sections, followed by loading on microscopic slides and staining with hematoxylin and eosin (H&E), Alcian blue, Safranin O, and Masson's Trichrome staining according to the manufacturer protocol. Images were captured with bright field microscopy (NiE, Nikkon, Japan). Further, histological scoring was determined and plotted. For immunohistochemical evaluation, alternate cryosections were used. Sections were fixed with 4% paraformaldehyde, followed by permeabilization, blocking, and staining with primary antibodies against AGGRECAN, SOX9, COlII, and TGFβ1. The immunohistochemical staining was visualized by treatment with secondary antibodies Alexa fluor 488 at a dilution of 1:200 for 1 h at 37°C. Nuclei were counterstained with DAPI for 10 min at RT. The tissue sections were visualized under a fluorescent microscope (NiE, Nikkon, Japan) to track Dil labeled hMSCs and chondrogenic markers expression. All the captured images were processed and quantified with Image J and plotted with MS excel.

### Histological grading

With slight modifications in the histologic grading system proposed in a prior study (31), thirty IVD sections comprising six sections in each group were graded blindly. The grading was performed based on structural changes, cellularity, and intensity of staining. The modified grading is presented in Table 2.

**Table 2:** Criteria for histological grading.

**Grading**	**Interpretation**
1	• Typical rounded NP and concentric rings of AF comprise 75% of cells • Uninterrupted borders between NP and AF • Well-organized collagen threads without ruptured or serpentine fibers • Deep stain in NP peri region and AF lamellae
2	• Rounded or irregularly shaped NP and AF comprise 75% of cells • Minimum interruption of the border between NP and AF • Slightly ruptured fibers in less than one-third of the transverse section • The pattern of stain is similar to grade 1, but with moderate fading from peri NP and AF region
3	• Atypically shaped NP and AF lamellae slightly decrease the number of cells in one-quarter to half of the disc • Well to moderate ruptured borders between NP and AF • Slightly ruptured fibers in more than one-third of the transverse section • Only one-third of the small area faded color either centrally or AF region
4	• Irregularly shaped NP and AF comprising cells < 75% of the half-quarter to half of the disc • Moderate interruption of the border between NP and AF • Moderately organized collagens lamellae with ruptured or serpentine fibers • Normal pattern no longer present, small area faded color centrally and in AF, but inconsistent and patchy
5	• Irregular NP and AF comprise < 50% of the cells • Sever interruption of the border between NP and AF • Rupturned or serpentine fibers in less than one-third of the transverse section • The stain completely washed off from the NP region

### Radiographic assessment

To attain a similar level of muscle relaxation of each animal at each time point during radiography, intense care was taken to maintain the sustained degree of anesthesia. Hence, preoperative radiographs were included as a baseline measurement. A non-de magnifying image intensifier with a collimator-to-film distance of 66 cm, penetration power of 30kV, and exposure of 40 mA of 40 s was used. The rats were laid in a prone position on a custom-made plastic slot plate, keeping their tail straight. Radiographs were scanned preoperatively and at 2 weeks after the index procedure. Radiographs were taken (LabScope, Glenbrook Technologies) and digitally stored *via* an image capture software program. The IVD height was calculated as the disc height index (DHI) based on the protocol mentioned by Masuda et al. (32). The disc height was averaged by measuring from the one-fourth, middle, and three-fourths area of the adjacent CEP width which was divided by the average of adjoining vertebral body height. Measurements were exported to Excel software, and IVD height was calculated as the disc height index (DHI = post-punctured DHI/adjacent IVD body height × 100) based on the previously developed method.

### Water content analysis

A total of 12 IVDs were dissected from rat tails for determining water content. Each disc was kept in pre-weighed vials, promptly weighed, and then kept in a heated oven for 24 h to determine dry weights *via* balance with ± 0.03 mg repeatability. Water content was evaluated as given below.


$$\%H_{2}O\;=\;100\%\;\times \;(wet\;weight-dry\;weight)/wet\;weight).$$

### Gag quantification

Dried IVD tissues were digested with papain at 58°C for 6 h, and total sulfated GAGs content was quantified using the 1, 9 dimethyl-methylene blue (DMMB) assay. Gag extracts in 96-well plates were allowed to react with DMMB at pH 3.1 (Sigma) and spectrophotometrically quantified at 525 nm. Total GAG was calculated from standard curves of chondroitin-6-sulfate C extracted from shark cartilage (Sigma).

### Gene expression analysis

At day 14, RNA was isolated from the IVDs tissue transplanted with transfected and non-transfected hMSCs for transcriptional evaluation. Primers for *Sox9, TGF*β*1, collagen type ll, aggrecan, BMP2, Six1, ADRB2, CXCL2, YKL40, Substance P, MMP-13, COMP2, SOD1, PRDX1, GPX1*, and *beta-actin (*β*-actin)* were used. The expression of the chondro-specific primers was normalized to β-actin expression. RNA from tissue samples was isolated by the Trizol isolation system (Invitrogen) and purity was determined with spectrophotomer. cDNA was prepared by RevertAidTM First Strand cDNA synthesis kit (K1622, ThermoScientific, USA) followed by qPCR amplifications using qPCR master mix (A600A, Promega, USA).

### Statistical analysis

Data were statistically analyzed using Microsoft Excel, and further assessment was done on GraphPad Prism5 software. All the data is presented as the mean ± standard deviation. For comparison between multiple groups, statistically significant differences between means were performed by one-way ANOVA followed by the Bonferroni *post hoc* test. For comparisons between the two groups, statistically significant differences between means were carried out by running an independent *T*-test, keeping *P* < 0.05 as a statistically significant difference.

## Results

### Characterization of human umbilical cord tissue-derived mesenchymal stem cells

**Figure 1:**
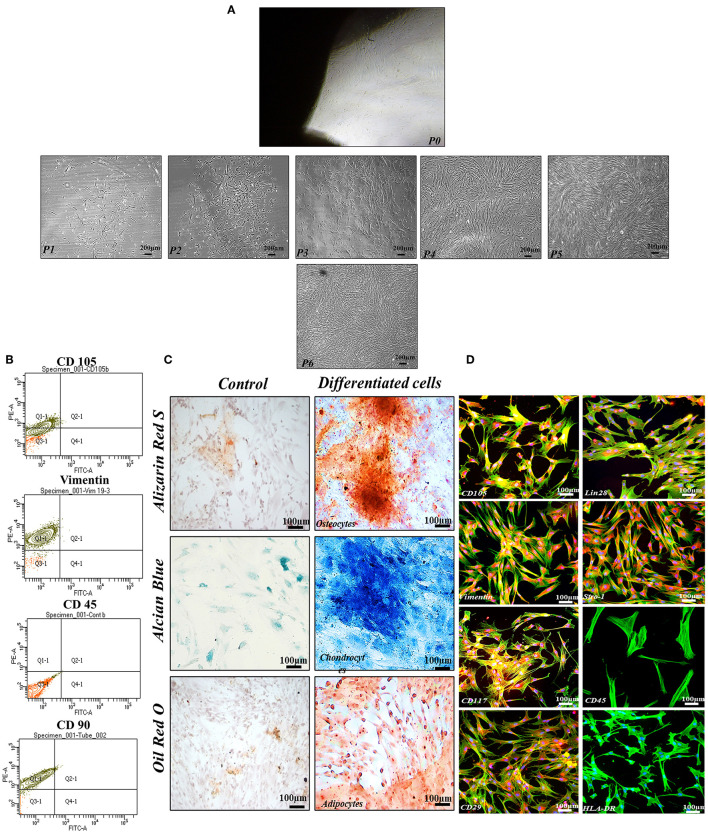
Functional and phenotypic characterization of hMSCs. **(A)** Showing the homogenous population exhibiting fibroblast-like morphology from P1 to P6. **(B)** Showing phenotypic characterization of hMSCs. **(C)** Functional characterization assay demonstrating that the hMSCs were successfully differentiated into osteocytes, chondrocytes, and adipocytes. **(D)** The cell showed positive expressions of MSCs specific markers including CD105, Vimentin, CD117, CD29, Lin28, and Stro-1whereas negative expression of CD45, and HLA-DR. F-actin and nuclei of the cell were visualized by phalloidin labeled with Alexa Flour 488, and DAPI. Imaging was performed using a fluorescent microscope at 10× magnification.

It has been observed that the cells released from the human umbilical cord tissue pieces exhibited homogenous, typical fibroblast-like morphology in the second to sixth passages with successful differentiation into chondrocytes, adipocytes, and osteocytes (Figures 1A, C). Herein, hMSCs showed positive surface expression of CD105, CD117, Vimentin, CD29, Lin28, and Stro-1 while negative expression of CD45, and HLA-DR with frequencies of Vimentin, CD105, and CD90 were observed to be >80%, in contrast, hMSCs that slightly expressed hematopoietic marker (CD45 < 15%; Figures 1B, D).

### Characterization of *Sox9* and *TFGβ1* transfected hMSCs

**Figure 2:**
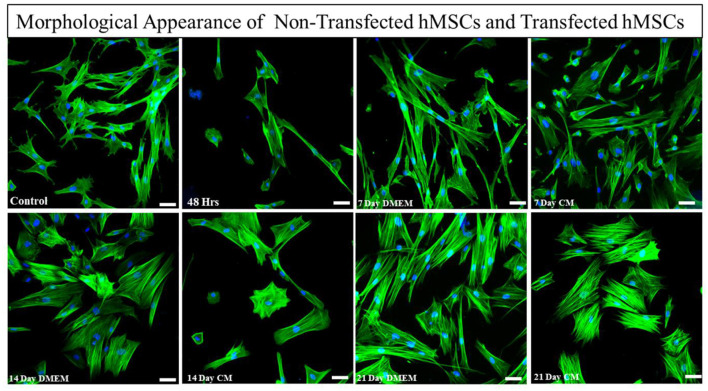
Morphological assessment of transfected and non-transfected hMSCs. Transfected hMSCs at 14 and 21 days of culture showed a broad, and polygonal appearance, whereas the control group exhibited fibroid-like morphology. Cellular cytoskeleton and nuclei were visualized by phalloidin labeled with Alexa Flour 488, and DAPI. Imaging was performed using a fluorescent microscope. Scale bar; 50 μm.

**Figure 3:**
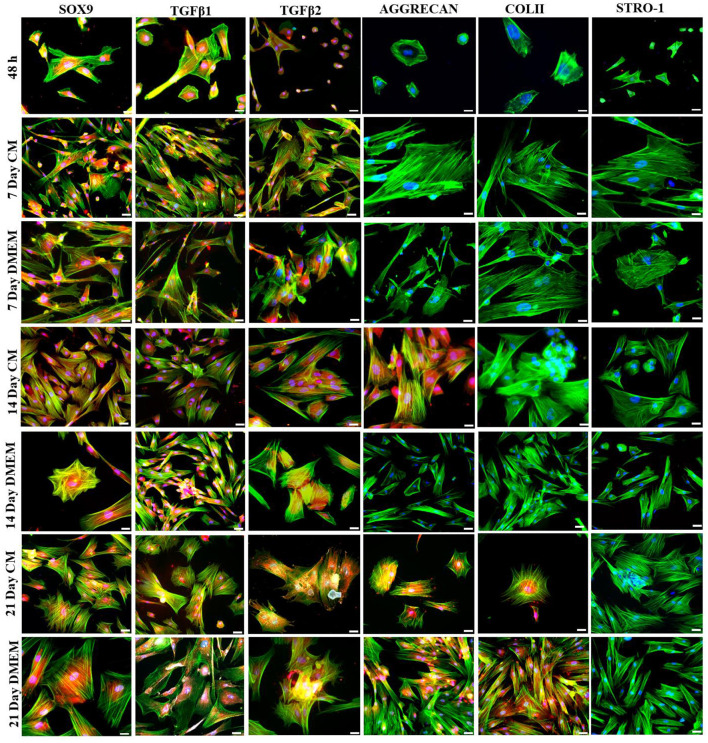
Transfection analysis based on immunocytochemical analysis. Transfected hMSCs at 48 h, 7 days in CM, and in DMEM, 14 days in CM, and in DMEM, and 21 days in CM, and DMEM, showed positive expression of SOX9, TGFβ1, TGFβ2, AGGRECAN, COLII and negative expression of STRO-1 under the fluorescent microscope at 20× magnification. The F-actin and nuclei were stained with phalloidin labeled with Alexa Four 488, and DAPI. Scale bar; 50 μm.

Transfected hMSCs showed remarkable differences in morphology in contrast to the hMSCs. A broad polygonal morphological feature was noted after 14 and 21 days of transfection (Figure 2). The transfected hMSCs after 48 h, 7, 14, and 21 days of culture in DMEM, and CM were immunostained for the expression of SOX9, TGFβ1, TGFβ2, AGGRECAN, ColII, and Stro1 as shown in Figure 3.

#### mRNA expression of chondrogenic genes after transfection of hMSCs

**Figure 4:**
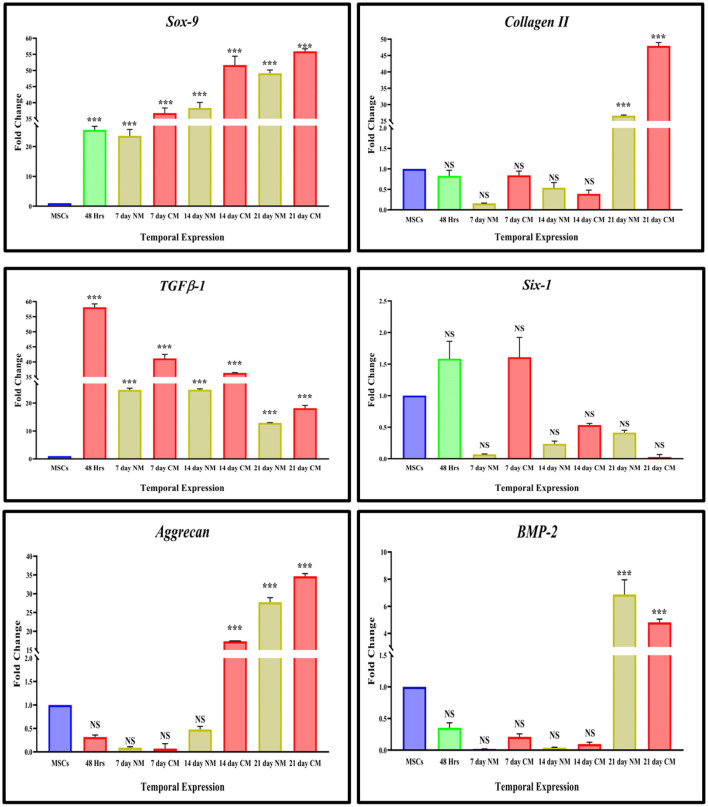
*In vitro* qPCR analysis. The bar graphs are demonstrating significantly elevated levels of *Sox9*, and *TGFB-1* at all the cultured time points. *ColII* and *aggrecan* were observed to be upregulated after 14 days of transfection. The expression of *Six1* was significantly downregulated in cells DMEM, and CM-cultured cells whereas *BMP2* showed significant upregulation at 21 days of culture. Data were statistically analyzed *via* the Bonferroni *post hoc* test. Values are presented as mean ± SD (*n* = 3). ***P < 0.001, NS, non-significant.

The expression of chondrogenic-related genes was examined by qPCR in both transfected and non-transfected groups (Figure 4). Detection of the early chondrogenic marker showed that the *Sox9* and *TGF*β*1* level was elevated at all the experimental time points, accompanied by an increased level of the late maker *aggrecan* and *ColII* after 14 and 21 days of culture, whereas expression of *Six1* was downregulated at all the time points. *BMP2* expression was elevated at 21 days of culture.

### Stemness analysis

**Figure 5:**
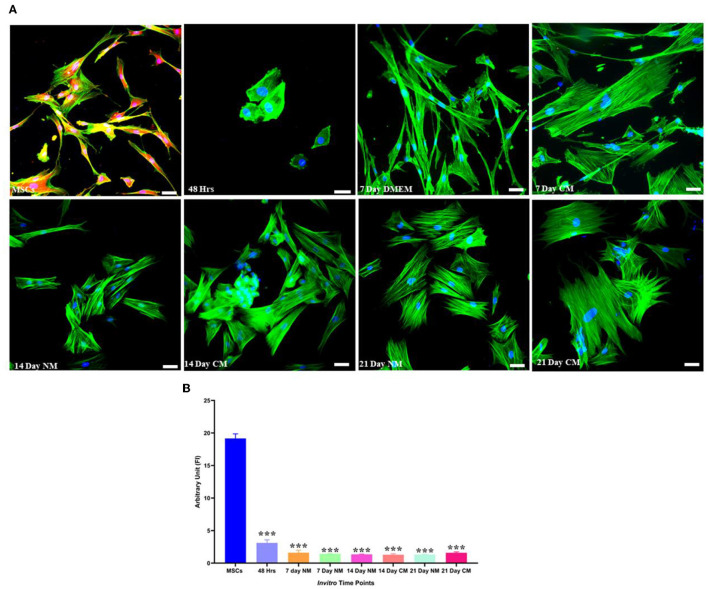
Stemness marker analysis. **(A)** Transfected hMSCs at 48 h, 7, 14, and 21 days of culture in CM and DMEM showed negative expression Lin 28 under the fluorescent microscope. The F-actin and nuclei were stained with phalloidin molecules of Alexa flour 488, and DAPI. **(B)** Illustrating the quantified expression of fluorescent intensities in contrast to the control group. Scale bar; 50 μm. ***P < 0.001.

Detection of stromal-specific markers showed that Lin28 was positively downregulated after the transfection, followed by a positive expression in non-transfected hMSCs (Figure 5).

### Histological assessment

**Figure 6:**
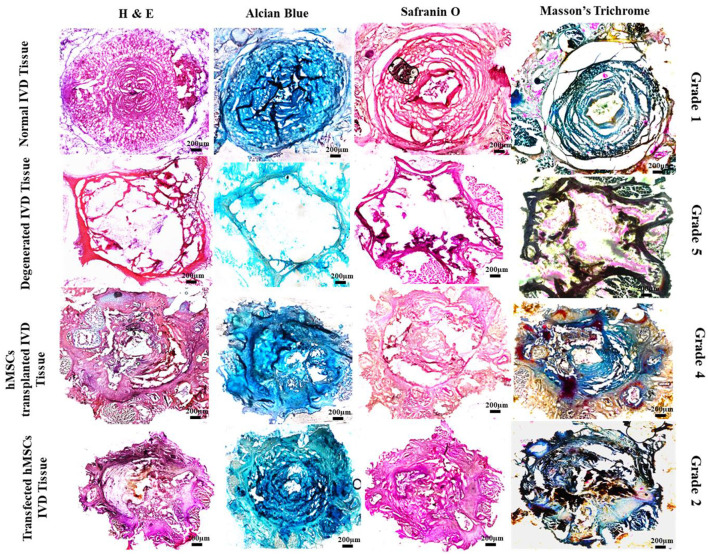
Histological assessment of treated and non-treated IVD. Histological assessment of normal IVD (Grade 1) showing intact central NP and concentric lamellae of AF-enriched cells, ECM and collagen threads (H & E, Alcian blue, Safranin O, and Masson's trichrome). The *Sox9* and *TGF*β*1* group (Grade 2) showed a similar pattern of structure and staining in contrast to the degenerated IVD (Grade 5) which exhibited poor staining and reduced regeneration of IVD. Degenerative ECM was characterized by empty space. Whereas the section examination of MSCs group (Grade 4) showed distorted regeneration of cartilage displaying slight production ECM and cracks between the collagen threads.

At 2 week-post of transplantation, the IVDD group (Grade 5) displayed a clearly disorganized infrastructure of the NP and a serpentine pattern of AF. The border between NP and AF was completely disrupted with a decreased number of chondrocytes. In contrast, *Sox9* and *TGF*β*1* group (Grade 2) exhibited a relatively well-restored NP, and distinct NP/AF border and chondrocytes-like cells in the region of NP. Safranin O and Alcian blue staining in the *Sox9* and *TGF*β*1* group displayed condensation of ECM-rich proteoglycan. The group showed relatively dark reddish-orange and blue staining. The group was highly similar to the normal IVD group (Grade 1), in contrast to the degenerated IVDD group completely washed-off stains in NP region were observed suggesting the reduced synthesis of proteoglycans. However, the MSC group (Grade 4), exhibited relatively faint staining of proteoglycans in NP compared to the *Sox9* and *TGF*β*1*, and normal IVD groups (Figure 6).

### Tracking and immunofluorescence assay

**Figure 7:**
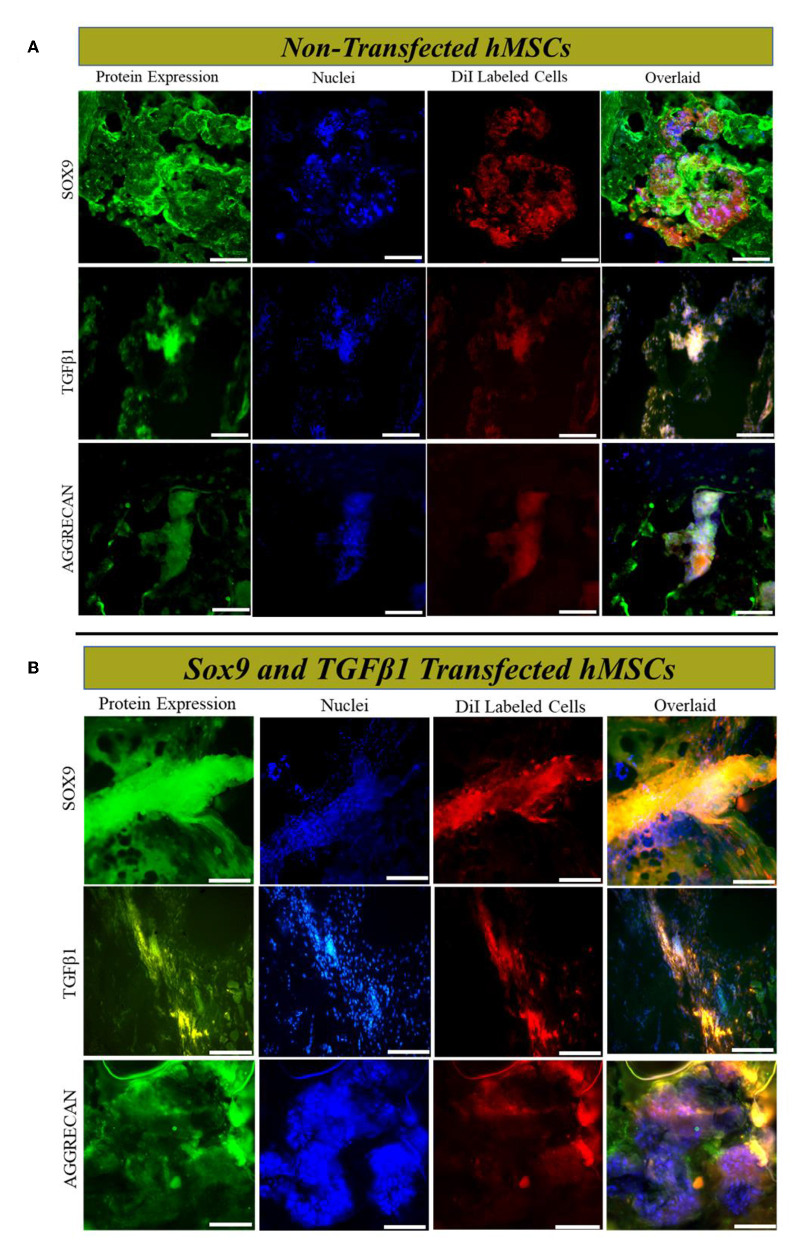
*In vivo* cells tracking and immunofluorescence analysis. Immunofluorescence staining **(A, B)** of rat IVD tissue transplanted with transfected and non-transfected hMSCs for SOX9, TGFβ1, and AGGRECAN, respectively. Sections were counterstained with DAPI. The yellow image is the overlaid image showing the co-localization of Dil-labeled cells with chondrogenic markers. Shown are the representative images of the staining of the tissues of IVD (*n* = 3) for each group. The images were captured using a fluorescent microscope at 20× magnification. Scale bar; 50 μm.

Dil-labeled cells were detected in the middle region of the MSCs, and *Sox9* and *TGF*β*1* group at 2 weeks of post-transplantation, illustrating the survival and homing of transplanted cells in IVD. Cells were Co-stained with SOX9, TGFβ1, and AGGRECAN, revealing that the *Sox9* and *TGF*β*1* transfected hMSCs cells expressed SOX9, TGFβ1, and AGGRECAN successfully *in vivo* (Figures 7A, B).

### Disc height index assessment

**Figure 8:**
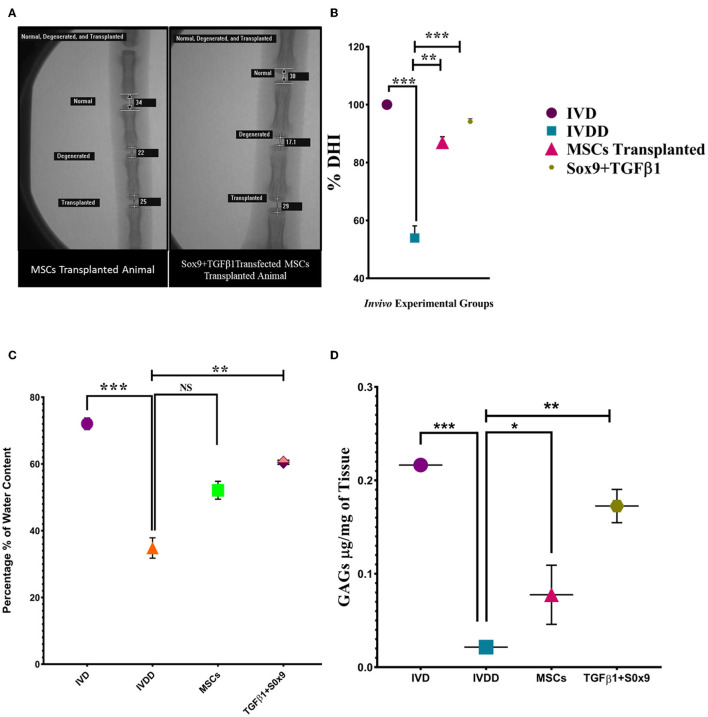
*In vivo* functional assay. **(A)** DHI after 2 weeks of post-degeneration, was found to be significantly decreased in the IVDD group compared to the normal IVD group. In the *Sox9* and *TGF*β*1* group, the disc height was markedly restored in contrast to the degenerated and MSCs group. The images were taken using a fluoroscopy system whereas the % DHI value **(B)** of the *Sox9* and *TGF*β*1* group at the index time was significantly upregulated than the MSCs group followed by a marked reduction in the IVDD group. **(C)** Water content was quantified by the heat evaporation method. The IVD transplanted with transfected hMSCs showed a significantly upregulated expression in contrast to the degenerated (IVDD) group. **(D)** The GAGs content of the *in vivo* group was quantified by the DMMB assay. The IVD transplanted with transfected hMSCs showed a significantly upregulated expression in contrast to the degenerated (IVDD) group. For statistical analysis, the Bonferroni *post hoc* test was performed. All the values are presented as mean ± SD (*n* = 3). ***P < 0.001, **P < 0.01, *P < 0.05, NS, non-significant.

Genetically modified hMSCs were transplanted into the NP region of the Co5–Co6 caudal disc. At 2 week post-transplantation, radiographs of the caudal disc were captured with the rats under anesthesia. Two weeks after transplantation, the mean % DHI of the transplanted disc in the genetically modified group was significantly higher than the degenerated (Co9–Co10) group and MSCs group. The differences in % DHI were significant at 2 weeks of post-transplantation (Figures 8A, B).

### Water and gag content evaluation

The water content of *in vivo* groups was quantified by a heat evaporation method. There was a significant marked decrease in water content in the IVDD group compared to the IVD. The quantified water content of the *Sox9* and *TGF*β*1* group was significantly higher than the IVDD and MSCs group (^**^P < 0.01; Figure 8C). GAGs analysis *via* DMMB assay enabled the detection of chondroitin sulfate, the major GAGs expressed in intervertebral discs. At significantly higher level of GAGs was detected in normal IVD and treated IVD when compared to degenerated IVD (^**^P < 0.01; Figure 8D). The DMMB finding supported the evidence of restoration of NP cells and GAGs.

### Transcriptional analysis of IVD tissue

**Figure 9:**
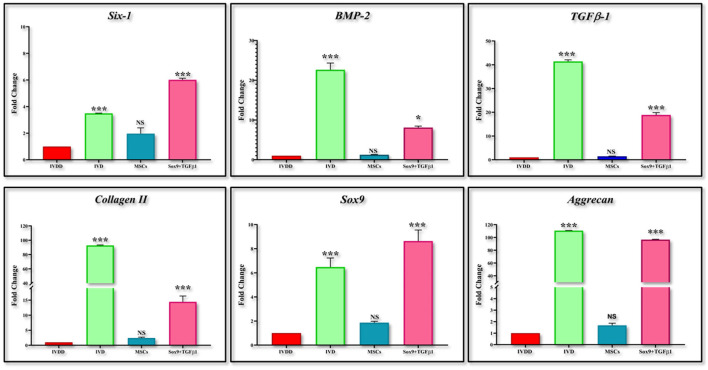
Gene expression analysis of chondrogenic markers. Bar graphs demonstrating the fold change in the mRNA expression of *Six-1, BMP-2, TGF*β*1, Collagen II, Sox9*, and *aggrecan* in group transplanted with transfected hMSCs and non-transfected hMSCs relative to the degenerated group by RT-PCR analysis. β*-actin* was used as a housekeeping gene. The group transplanted with transfected hMSC was significantly upregulated than the degenerated group. The *Y*-axis is demonstrating the fold change. The fold change of degenerated group is set at one. For statistical analysis, the Bonferroni *post hoc* test was performed. All the values are presented as mean ± SD (*n* = 3). ***P < 0.001 and *P < 0.05. NS, non-significant.

**Figure 10:**
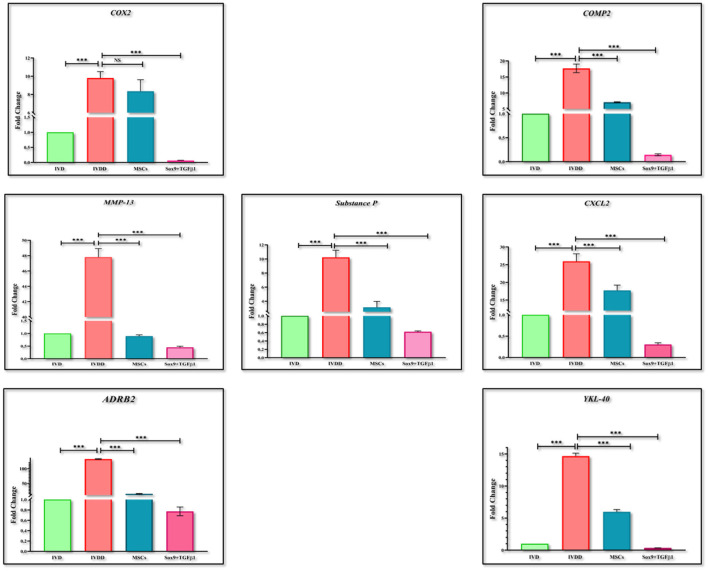
Gene expression analysis of pain and inflammatory markers. Bar graphs demonstrating the fold change in the mRNA expression of *COX2, MMP-13, ADRB2, Substance P, COMP2, CXCL2*, and *YKL-40* in group transplanted with transfected hMSCs and non-transfected hMSCs relative to the degenerated group by RT-PCR analysis. β*-actin* was used as a housekeeping gene. The group transplanted with transfected hMSCs showed significantly lower expression than the degenerated group. The Y-axis is demonstrating the fold change. The fold change of normal IVD is set at one. For statistical analysis, the Bonferroni *post hoc* test was performed. All the values are presented as mean ± SD (*n* = 3). ***P < 0.001, NS, non-significant.

**Figure 11:**
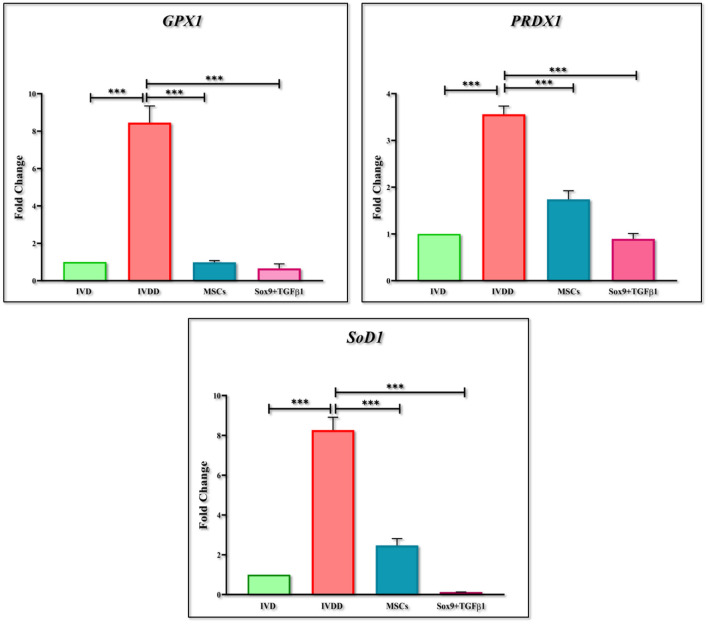
Gene expression analysis of oxidative stress markers. Bar graphs demonstrating the fold change in the mRNA expression of *GPX1, PRDX1* and *SOD1* in group transplanted with transfected hMSCs and non-transfected hMSCs relative to the degenerated group by qPCR analysis. β*-actin* was used as a housekeeping gene. The group transplanted with transfected hMSCs showed significantly lower expression than the degenerated group. The Y-axis is demonstrating the fold change. The fold change of normal IVD is set at one. For statistical analysis, the Bonferroni *post hoc* test was performed. All the values are presented as mean ± SD (*n* = 3). ***P < 0.001.

RT-PCR was performed for the estimation of chondrogenic genes involving *Sox9, TGF*β*1, Aggrecan, ColII, Six1*, and *BMP2*, pain, and inflammatory genes involving *COX2, COMP2, MMP-13, ADRB2, CXCL2, YKL40*, and *Substance P*, and OS genes involving *PRDX1, GPX1*, and *SOD1*. The findings indicated that the expression of chondrogenic genes was upregulated in the group transplanted with transfected hMSCs compared to the IVDD group (Figure 9). However, the expression of pain and inflammatory genes (Figure 10), and OS genes (Figure 11) showed a positive downregulation in the group transplanted with transfected hMSCs compared to the IVDD group at 14 days of post-transplantation (^***^P < 0.001). These data revealed that Sox9 and TGFβ1 transfected hMSCs can enhance chondrogenesis and reduce pain and inflammation, and OS in the degenerated IVD.

## Discussion

The global healthcare target is to reverse the pathology and maintain the native IVD tissue to reduce its impact on daily health and the economy (33). The degenerated intervertebral disc (IVDD) is one the most challenging clinical problem as it majorly results in disability among the aged population (34). It is important to comprehend the actual causes of degeneration before determining a therapeutic strategy. The definite cause of disc degeneration has yet not been identified however, various factors have been documented including poor nutrition, reduced biomechanical stability, genetic factors, and mechanical stress (35). The primary obstacle in developing a regenerative therapy for degenerated IVD is due to its highly complicated structure and stringent environment. Disc degeneration is associated with reduced viable chondrocytes or NP cell death and less proteoglycan content within the extracellular matrix followed by dehydration (36). As the degeneration progresses, the capability of IVD to support body load and flexion becomes compromised. Eventually, it becomes unstable, followed by severe discogenic pain. The demand for the development of specific therapeutic approaches to enhance IVD healing, and the regeneration process is evident that current therapies have glitches in the treatment of such debilitating diseases (37). Therefore, to overcome these glitches, genetically modified stem cell is proven to be effective as they can potentially replicate IVD homeostasis leading to natural tissue repair (38).

The findings reported in this study corroborate the significant role of the combinatorial effect of transcriptional regulators in the regulation of aggrecan and type 2 collagen production. The authors have developed a novel combination of *Sox9* and *TGF*β*1* transcription regulators and tested them on human umbilical-derived mesenchymal stromal cells. Exogenous MSCs are immune privileged, they are self-proliferative which offsets the repair of degenerated IVD (39). These MSCs may have the ability to differentiate into functional chondrocytes and enhance the synthesis of aggrecan and type 2 collagen to repair the degenerated cartilage *via* paracrine factors, which is useful to reverse the pathology (40). Therefore, in present study, MSCs were isolated from human umbilical cord tissue as cord tissue is rich in MSCs which possessed immense pluripotency and were identified as expressing a series of surface markers including CD105, CD117, CD29, CD90, CD70, Vimentin, Stro-1, and Lin28. They were capable to differentiate into lineages of mesenchymal tissue including adipose tissue, bone, and cartilage. This identification has been previously illustrated in the literature (41–47). The overexpressed chondrogenic transcriptional regulators in hMSCs increased the production of *Sox9, TGF*β*1*, and *Six1* after 48 h of transfection upregulated *aggrecan* expression at day 14 by interacting with the promoter region. This enhanced the *Type 2 collagen* synthesis after 3 weeks of transfection at the cellular level *in vitro*. These consequences are supported by the Ouyang et al., investigation in which human chondrocytes were transduced with the pLv-CMV-SOX9 lentiviral plasmid which resulted in the marked increase of *ColII, aggrecan, TGF*β*1*, and *Smad3* in an osteoarthritis model of mice. With the increased level of Smad3, mRNA expression of inflammatory markers including MMP-13, and IL-1β was significantly reduced (48).

Furthermore, for a rat intervertebral disc degeneration model, preliminary data emerged to divulge that injection of genetically modified hMSCs after inducing injury significantly increased the GAGs content which is a typical biochemical property of IVD. This outcome was likely driven by a higher count of cells resulting in the restoration of NP. This is in consensus with the synthesis of GAGs detected by the Dimethyl methylene blue (DMMB) assay in prior degenerative models (49, 50). The transplanted cells rejuvenated the IVD infrastructure (H & E) comprising the nucleus pulposus and annulus fibrosus. This culminates the glycosaminoglycan GAGs production over the period of 2-weeks. The crisp (Alcian blue and Safranin O) staining on acidic proteoglycan deposits in the NP demonstrated that this could be a rescued behavior. Additionally, the heterogeneously aligned collagen threads (Masson's trichrome) in the normal and treatment group supported the identification of a significant increase in collagen production which is in consensus with the study performed on rabbit models. Analogous outcomes were corroborated by the erstwhile literature (51, 52). The histological grading was modified consisting of grade 1 (normal IVD) to grade 5 (degenerated IVD). Our study revealed the severe disruption of NP and highly fibrotic lamellae, with optically empty from the center resulting in clear washed-off stains signifying the degeneration consecutive to consistent degenerative response (grade I). At grade II, the structure displayed similar structural integrity as grade I with negligible disruption of the NP and AF border. The tissue section transplanted with genetically modified cells was categorized as grade II, confirming the complete regeneration of the tissue. Grade IV is the middle stage of regeneration. Irregular NP and AF integrity, with moderate interruption of NP/AF borders and collagen fibers exhibiting the partial restoration of the IVDs structure by MSCs at day 14 of post-transplantation (31, 53). Therefore, this could go with the outcomes stating that the biological repair by genetically modified hMSCs in the ongoing degenerated environment can ideally be achieved by administering them at the early stage of degeneration.

There is potent evidence from x-ray and MRI studies indicating that lower back pain is a well-established condition arising at the early stages of disc degeneration and thus it is linked with spinal cord injury which has a pronounced inverse effect on disc height. The reduction of disc height is the key pathological manifestation of the degeneration which can be detected by radiographic images (54, 55). This is supported by Chen et al., a study in which the authors investigated the intra- and inter-rater reliable protocol of analyzing disc height index (DHI) by taking radiographs following a structured procedure of x-ray scan. The full penetrative puncture was radiographically assessed *via* X-ray, which showed significant disc space narrowing in the degenerative group (IVDD) in contrast to the normal group IVD, and cell transplanted group. This supported the phenomena of the successful retention, and differentiation of cells, and restoration of the native IVD homeostasis (56).

To typify the chondrogenic composition of nucleus pulposus throughout the period of experimental time point, we investigated the mRNA expression of *Six1, Sox9, TGF*β*1, BMP2, aggrecan*, and *colII* of the transplanted cells after 2 weeks of puncture by 18G needle. *Sox9* possesses transcriptional action in promoting chondrogenesis as an activator of cartilage-liked genes. Being the master regulator of chondrocytes (57), it works efficiently with downstream and upstream of the other regulators and potentially hit the expression of *aggrecan* and *colII* (58). As a structural component, *aggrecan*, and *ColII* help in mediating chondrocyte-chondrocyte and chondrocyte-ECM interaction (59, 60) unveiling a burst increase in *aggrecan* and *ColII* mRNA expression on the day of harvest. *TGF*β*1*, along with *Sox9* cooperate in *Sox9* transactivation.

Together, these genes proved that overexpression of *Sox9* and *TGF*β*1* may directly target distinct gene enhancers at various cell differentiation levels and in various cartilage types to account for temporal gene expression such as gene encoding key cartilage-liked ECM genes (*link protein, collagen type II, XI, IX*, and *aggrecan*), the initial regulators (*Six1* and *BMP2*) (61–63).

The experiment utilizing chondrogenic antibodies against labeled cells were also investigated based on the specific interaction of antigen-antibody complex to analyze the localization, homing, and protein distribution of transplanted cell in tissue which validated the qPCR outcomes.

IVD inflammation, pain and its relationship to cartilage destruction is a topic of debate (64). The examination at the transcriptional level has resulted in a further understanding of the degradation cascade in the diseased models. Serum biomarkers for cartilage degradation due to inflammation and oxidative stress (OS) have been investigated primarily in the IVDD model of rabbits (65, 66). However, the exhaustion of proteoglycans and water content, and reduction in the disc height in association with increased inflammatory and oxidative stress profile have not been clearly understood. Since, *Sox9*, and *TGF*β*1* together participate in various vital processes, such as the development, proliferation, differentiation, and suppression of non-chondrocyte elements. Due to their regulatory roles in biological processes, their expression must be tightly controlled. The impact of the *Sox9* and *TGF*β*1* on IVDD progression revealed that elevation of *Sox9* and *TGF*β*1* was able to repress inflammatory and oxidative stress response in IVDD (67). Reactive oxygen species are highly reactive and unstable molecules. They include hydrogen peroxide (H_2_O_2_), hydroxyl radical (OH^−^), superoxide ions (O${}_{2}^{-}$), and hypochlorite ions (OCL^−^). As discussed earlier, IVD has a low nutritional supply, however NP, and AF cells are aerobic. During disc degeneration, due to NP cell death, an increase in blood supply stimulates ROS accumulation resulting in oxidative stress. Oxidative stress contributes to the imbalance of antioxidants and ROS production leading to cellular death, ECM degradation, and increased inflammatory cytokines accumulation (68). Therefore, patterns of *ADRB2, CXCL2, YKL40, Substance P, MMP-13, COMP2, PRDX1, GPX1*, and *SOD1* markers were also evaluated. The higher levels of *ADRB2, CXCL2, YKL40, MMP-13*, and *COMP2* in the IVDD group, and significantly lower levels in cell-transplanted groups indicated an improvement in degenerative changes. The reduced level of *COMP2* reflected the increased production of ECM which is in accordance with the results reported by Stellavato et al. (69). The presence of discogenic pain caused by a change in the expression pattern of pain-related peptide *substance P* was also examined (70). The downregulation of *substance P* level in the *in vivo* group injected with transfected hMSCs indicated the abatement of back pain which demonstrated that this could be a promising treatment for discogenic pain. Hence, downregulation in the inflammation and pain markers by genetically modified cells could be considered as a salvage behavior in the degenerative environment. These outcomes were found to be consistent with a prior study, demonstrating that chondrocyte transplantation was potentially an attempt to reduce inflammation, avoid proteoglycan loss, and restore disc integrity (71, 72). Following an increase in the expression of *PRDX1, GPX1*, and *SOD1* in the IVDD group at 2 weeks post-transplantation in contrast to the cellular transplantation group, explains by dysfunctional damage of NP, and cellular exhaustion (73).

Studies suggested that the suppression of such response during disc degeneration is due to the inhibition of NF-κB, thereby decelerating the development of IVDD (74–76). The potential of NF-κB inhibition to reduce cartilage degeneration has been previously described and the p-p65 termination revealed the inactivation of NFκB, which contributed to suppressing the development of IVDD (77).

Moreover, variations in water content were also interrogated. The findings revealed that genetically modified cells were found to be effective in the complete recovery of disc hydration. Zhu et al. compared the consequences of different therapies in restoring the water content of the human lumbar disc. The result was in line with our finding which proved that cellular therapy successfully recovered the water content (78, 79). Therefore, therapies enhancing the GAGs production or reducing the GAGs degradation are effective in restoring the water content and disc height index.

The major outcome of the study is that the transplantation of transfected hMSCs in the harsh degenerative microenvironment of the disc, restored the normal tissue architecture, and these cells homed, survived, and integrated into the disc. They efficiently revitalized the regeneration cascade and improved genomic stability by quenching ROS levels resulting in the decline of inflammatory and pain markers. The accelerated chondrogenesis of hMSCs can be achieved by co-transfection of *Sox9* and *TGF*β*1* resulting in the cure of the manifestations associated with intervertebral disc degeneration. Therefore, it is convincible that the forced expression of *Sox9* and *TGF*β*1* can be adopted as a novel therapeutic strategy to enhance the potential cartilage tissue engineering with better restoration of normal disc. This study established the basis for clinical investigation and translation of genetic modification approach for the regeneration intervertebral disc diseases.

## Data availability statement

The raw data supporting the conclusions of this article will be made available by the authors, without undue reservation.

## Ethics statement

The studies involving human participants were reviewed and approved by Independent Ethical Committee. The patients/participants provided their written informed consent to participate in this study. The animal study was reviewed and approved by Dr. Panjwani Center for Molecular Medicine and Drug Research University of Karachi.

## Author contributions

SK performed the experiments and wrote the manuscript. SE and FR helped in experimentation. AS evaluated the data and helped in writing. IK designed the experiments, analyzed the data, secure the funding, and finalized the manuscript. All authors contributed to the article and approved the submitted version.
